# An open label randomized multicentre phase IIIb trial comparing parenteral substitution versus best supportive nutritional care in subjects with pancreatic adenocarcinoma receiving 5-FU plus oxaliplatin as 2^nd ^or higher line chemotherapy regarding clinical benefit - PANUSCO

**DOI:** 10.1186/1471-2407-9-412

**Published:** 2009-11-27

**Authors:** Angela Märten, Moritz N Wente, Jennifer Ose, Markus W Büchler, Ingeborg Rötzer, Christiane Decker-Baumann, Irini Karapanagiotou-Schenkel, Sabine Harig, Jan Schmidt, Dirk Jäger

**Affiliations:** 1 University of Heidelberg, Department of Surgery, Im Neuenheimer Feld 110, 69120 Heidelberg, Germany; 2 National Center for Tumor Diseases, Im Neuenheimer Feld 350, 69120 Heidelberg, Germany

## Abstract

**Background:**

Pancreatic cancer is an extremely aggressive malignancy. Subjects are afflicted with a variety of disconcerting symptoms, including profound cachexia. Recent data indicate that the outcome of oncological patients suffering from cancer cachexia could be improved by parenteral nutrition and that parenteral nutrition results in an improvement of quality of life and in prolonged survival.

Currently, there is no recommendation of routine use of parenteral nutrition. Furthermore, there is no clear recommendation for 2^nd ^line therapy (or higher) for pancreatic adenocarcinoma but often asked for.

**Methods/Design:**

PANUSCO is an open label, controlled, prospective, randomized, multicentre phase IIIb trial with two parallel arms. All patients will be treated with 5-fluorouracil, folinic acid and oxaliplatin on an outpatient basis at the study sites. Additionally, all patients will receive best supportive nutritional care (BSNC). In the experimental group BSNC will be expanded with parenteral nutrition (PN). In contrast, patients in the control group obtain solely BSNC. Parenteral nutrition will be applied overnight and at home by experienced medical staff.

A total of 120 patients are planned to be enrolled. Primary endpoint is the comparison of the treatment groups with respect to event-free survival (EFS), defined as the time from randomization till time to development of an event defined as either an impairment (change from baseline of at least ten points in EORTC QLQ-C30, functional domain total score) or withdrawal due to fulfilling the special defined stopping criteria for chemotherapy as well as for nutritional intervention (NI) or death from any cause (whichever occurs first).

**Discussion:**

The aim of this clinical trial is to evaluate whether parenteral nutrition in combination with defined 2^nd ^line or higher chemotherapy has an impact on quality of life for patients suffering from pancreatic adenocarcinoma.

**Trial registration:**

Current Controlled Trials ISRCTN60516908.

## Background

Pancreatic cancer is an extremely aggressive malignancy characterized by extensive invasion, early metastasis, and marked cachexia. The yearly incidence of pancreatic cancer overlaps the mortality rate and the 5-year survival rate is less than 5% [1]. Subjects are afflicted with a variety of disconcerting symptoms, including profound cachexia and deterioration in performance status (PS), even when their tumour burden is low [2,3]. Therefore, one of the most important therapeutic targets is the improvement of quality of life (QoL) [4].

Cachexia is a strong independent predictor of mortality, poor therapeutic response, diminished functional capacity, and reduced QoL. It is defined as the debilitating state of involuntary weight loss, often connected with anorexia, tissue wasting, malnutrition, and inability for natural nutrition intake. The combination of these symptoms is also named "cancer anorexia-cachexia syndrome" [4]. The most important phenotypic feature is muscle wasting and functional impairment as a result of increased protein degradation and reduced protein synthesis or both [5]. In addition, it is a life-threatening and debilitating syndrome for 50% of all cancer subjects [5]. Compared to other solid tumours, the pancreatic cancer has the highest incidence for cancer cachexia (CC), as much as 80% at the time of diagnosis [4].

It has to be considered that the full effect of CC can not be identified by weight loss alone. Reduced food intake and systemic inflammation are also involved. Therefore, a multifactor cachexia profile was developed by the group of Bauer et al., based on weight loss, food intake and inflammatory status. These variables were investigated by determination of dietary intake, body composition, nutritional status, functional capacity and QoL in subjects with CC receiving chemotherapy [6].

As reported, the QoL is significantly impaired in CC subjects and there is evidence for increased morbidity and mortality of subjects with CC [5]. Subjects suffering from CC commonly have derangements in basal metabolic rate as well as reduced appetite and food intake. Even moderate weight loss is associated with psychological stress and lower QoL. Furthermore, CC affects subject's surgical risk and response to first- and second-line chemo-/radiotherapy [5]. Cachectic subjects are difficult to guide through chemotherapy experiencing more severe side effects resulting in progressive cachexia. The outcomes of subjects with CC receiving chemotherapy can be improved parenteral nutrition (PN) [6]. Supplementation with PN improves the QoL in subjects with advanced CC [7]. There are also data indicating that PN has a synergistic effect with chemotherapy and results in better clinical outcome [6,7]. Further research in form of randomized controlled trials is required to confirm these results. Besides, it is important to establish criteria for starting PN in cancer subjects [8]. The European Society of Parenteral and Enteral Nutrition (ESPEN) recommend PN only for malnourished subjects but does not reflect situation in CC patients. Furthermore, there is no recommendation of routine use of PN during chemotherapy, radiotherapy or a combined therapy [5,9].

At present, no 2^nd ^line therapy (or higher) is recommended for pancreatic adenocarcinoma, but often asked for [10]. In this clinical trial 5-FU (5-fluorouracil), folinic acid (FA) and oxaliplatin will be given as chemotherapy according to the OFF-scheme developed in the context of the clinical trial CONKO-003 [11]. This trial proofed a clinical benefit of 5-FU plus oxaliplatin as 2^nd ^line chemotherapy for subjects suffering from pancreatic adenocarcinoma. The reported median Overall Survival (OS) of 5 months is significantly higher than in the control group receiving 5-FU and FA (4.1 month; *p *= 0.014). Data from literature report median OS ranging from 4.3 month to 6.5 month [12,13]. Nevertheless, more scientific work is required to show that 2^nd ^line chemotherapy especially in combination with PN can improve the QoL in subjects suffering from pancreatic cancer.

## Methods/Design

### Trial organization

PANUSCO is designed and coordinated by the Department of Surgery, University of Heidelberg and the National Center for Tumor Diseases (NCT) in Heidelberg. Heidelberg is responsible for overall trial management, regulatory affairs, statistical planning and analysis, trial registration (International Standard Randomised Controlled Trial Number (ISRCTN60516908), http://www.controlled-trials.com) and reporting as well as quality assurance. The responsibility for external monitoring and pharmacovigilance is carried forward to independent Contract Research Organisations (CRO). The trial will be performed at four University Hospitals in Germany. The trial is sponsored by the foundation "Leben mit Krebs" in Germany. The financial sponsor is not involved in the data base management and has no access to the randomisation code.

### Investigators

Patients will be recruited by the four planned study sites. Special contracts will be disposed with the involved study centres. Due to the multimodal nature of the trial, all investigators will either be experienced oncologists or gastroenterologists. Additional, for the NI experienced nutritionists will take care for PN and BSNC.

### Data Safety Monitoring Board

An independent Data Safety Monitoring Board (DSMB) consisting of three experts (one expert in pancreatic carcinoma, one expert in nutritional medicine and one expert in biostatistics) will evaluate the clinical research data on an ongoing basis to assure patient safety and study integrity for the clinical trail. The board will monitor the trial data, in particular the safety data, and makes recommendations based on the periodically reviewed data. Responsibilities are laid down in a DSMB Charta.

### Medication supply

All chemotherapeutic agents will be prepared and provided by the corresponding pharmacy. Chemotherapeutic medication will be prepared for each patient specifically and delivered just prior to administration to the outpatient's department. PN will be applied overnight at patient's home by medical-experienced staff. Therefore, PN will be prepared at pharmacies next to the patient's home.

### On-site monitoring

Monitoring will be done by personal visits from clinical monitors according to the Standard Operating Procedures (SOPs) of the independent, external CRO. Monitors will review the entries into CRFs on the basis of source documents (30% source data verification). Data management will be performed by the Institute of Medical Biometry and Informatics (IMBI) at the University of Heidelberg. Pharmacovigilance is outsourced to a further CRO specialized on safety issues. Both data bases will be aligned at the end of the trial.

### Ethics, informed consent and safety

The final protocol was approved by the ethics committee of the University of Heidelberg, Medical School at September 22^nd^, 2009. The clinical trial complies with the Helsinki Declaration from 2008, the Medical Association's professional code of conduct, the principles of Good Clinical Practice (GCP) guidelines and the Federal Data Protection Act. The trial will also be performed in keeping with local legal and regulatory requirements. The medical secrecy and the Federal Data Protection Art will be followed.

Written informed consent for the clinical trial PANUSCO and respectively separate informed consent for the accompanying translational research will be obtained from each participating patient in oral and written from before inclusion in the trial. The nature, scope and possible consequences of the trial have been explained by a physician in detail. The investigator will not undertake any measures for the clinical trial or the translational research until valid consent has been obtained.

### Patient selection

PANUSCO focuses on hospitalised patients over 18 years of age suffering from advanced pancreatic adenocarcinoma with previous progression under chemotherapy.

Men and women over 18 years with histological confirmed advanced pancreatic adenocarcinoma which have received at least one previous chemotherapy (gemcitabine-based) will be screened for participation in the clinical trial. Patients will be contacted first-time by the (sub-) investigators at the outpatient's department. A detailed overview of recruitment criteria for inclusion and exclusion is given in table 1.

**Table 1:** Eligibility Criteria

Inclusion criteria	Exclusion criteria
Written informed consent (IC)	Major surgery < 4 weeks prior to enrolment
Histological confirmed advanced pancreatic adenocarcinoma	Weight loss > 2% within the last seven days or caloric intake ≤ 500 kcal expected within the next five days
At least one previous chemotherapy (gemcitabine-based)	PINI-Index > 10
≥ 18 years old	Pregnancy or breastfeeding
Body weight ≥ 50 and ≤ 95 kg	> 4 weeks of parenteral nutrition within the last 6 months
BMI ≥ 19	Parenteral nutrition < 4 weeks prior to enrolment
Negative pregnancy test (females of childbearing potential)	Vulnerable populations (e. g. subjects incapable of giving consent personally)
Willingness to perform double-barrier contraception during study	Subject selection conflicts with warnings, precautions and contraindications stated for any investigational product
Expected life expectancy > 3 months	

### Study design

**Figure 1:**
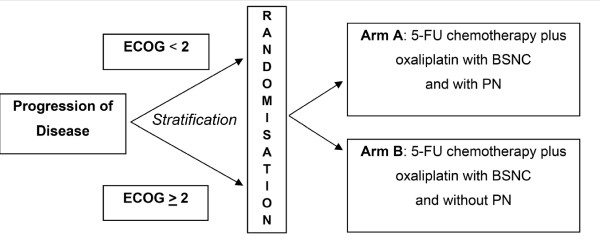


PANUSCO is an open label, controlled, prospective, randomized, multicentre phase IIIb trial with two parallel study arms (Figure 1). All Patients receive chemotherapy (a combination of 5-flourouracil (5-FU), folinic acid (FA) and oxaliplatin) at an outpatient's basis at the study sites. Additional, the patients in experimental group receive BSNC expanded with PN containing a total of 1150 kcal for each application. PN will be given by medical-experienced staff overnight at patients home. The control group receives solely BSNC consisting of nutritional advice. Every kind of enteral nutrition and oral supplementation is allowed for patients in both study arms.

### Study objectives

Primary objective is the comparison of the treatment groups with respect to event-free survival (EFS). EFS is defined as the time from randomization till time to development of an event defined as either an impairment (change from baseline of at least ten points in EORTC QLQ-C30, functional domain total score) or withdrawal due to fulfilling the special defined stopping criteria for chemotherapy as well as PN and Best Supportive Nutritional Care (BSNC) in Arm A or BSNC alone in Arm B or death from any cause (whichever occurs first). Chemotherapy will be continued until individual stop-criteria (disease progression, unacceptable toxicity) are met. This is unaffected by the (dis-) continuation of NI in both study arms. NI in both arms will be stopped when two of the three following criteria are met:

1. weight loss > 2% within the last seven days or caloric intake ≤ 500 kcal expected within the next five days,

2. BIA (Bioimpedance Analysis) defined by phase angle and body cell mass (BCM) with a deterioration > 10% (in both parameters)

3. Prognostic Inflammatory and Nutritional Index (PINI) > 10 (only in subjects with no sign of acute inflammation).

This is unaffected by the (dis-) continuation of chemotherapy.

Secondary objectives are the comparison of the treatment groups with respect to tumour-cachexia, Objective Response Rate (ORR), Time to Progression (TTP), Progression Free Survival (PFS), Overall Survival (OS), Toxicity, time from randomization till time point when stopping criteria are met. Furthermore, the definition and evaluation of a scoring system identifying subject groups who will benefit from second line chemotherapy and/or PN.

Accompanying translational research is focussing mainly on molecular signalling as well as on inflammatory parameters. The available results will be correlated with the clinical parameters.

### Randomisation and standardised treatment scheme

All patients enrolled will be identifiable throughout the study. The investigator will maintain a personal list of patient numbers and patient names to enable records to be found at a later date. Upon inclusion each patient receives a unique identification number. After the patient's eligibility for randomisation has been assessed he/she will be randomly assigned to one of the two treatment arms (1:1) and receives a unique randomization number. The randomisation will be done and documented centrally and send by fax. Stratification will be performed prior to randomization by Eastern Cooperative Oncology Group Performance Status (ECOG PS) (stratum 1: PS < 2, stratum 2: PS ≥ 2). The randomisation list will be kept in safe and confidential custody at the involved CRO. The method of randomisation and the block sizes themselves will not be disclosed to the study sites, the monitors or any person involved in the clinical trial conduct. This information will be kept with the randomisation list.

### Treatment scheme

After implantation of a catheter patients will be treated as follows:

Both study arms receive a chemotherapy consisting of 2000 mg/m^2^/day 5-FU by IV (24 hours) 200 mg/m^2^/day FA IV (30 min) on day 1, 8, 15 and 22 together with 85 mg/m^2^/day oxaliplatin IV (2 hour) on day 8 and 22. Therapy will be interrupted between days 23 to 42. This cycle will be repeated until any stop-criterion for chemotherapy (disease progression or unacceptable toxicity) is met.

Furthermore, all patients receive nutritional consultation and recommendation by experienced nutritionist contemplated as BSNC.

#### Experimental group

Additionally, patients in experimental group receive a total amount of 1036 ml PN, applied over night via port system. The PN will be administered continuously six days a week. An interruption will be performed during chemotherapy. Beyond, an adjournment of the PN up to two days is possible; an adjournment of three days is only once possible during participation of the clinical trial.

### Evaluation, toxicity-based dose adjustment and follow-up

All patients must have appropriate lab and radiographic studies conducted prior to study enrolment to meet eligibility criteria. Lab parameters will be obtained at least weekly and imaging will be performed every seven weeks. The patient will be asked at each visit for any adverse event (AE) as well as concomitant medication and pain medication. Quality of life (QoL) questionnaires (QLQ-C30 and the pancreas specific PAN 26 questionnaire) will be handed out to all patients on day 1 and day 22 of each cycle prior to chemotherapy. Decisions regarding weekly chemotherapy treatment and chemotherapy dose-adjustment will be made using the guidelines below and based on haematological parameters monitored once weekly during chemotherapy. In case of grade 3 or grade 4 haematological toxicities chemotherapy should be held until resolution or until toxicity has resolved or dropped to grade 2. PN will be continued. The procedures for patients with leukocytes between 1.0/l and 1.5/l will be discussed individually in the study group. In case of grade 3 or grade 4 gastrointestinal toxicities (including anorexia, dehydration, nausea/vomiting, diarrhoea, mucositis and bleeding) patients will be considered for periodic outpatient intravenous rehydration with anti-emetics according to conventional practice guidelines. Any grade ≥ 2 neurotoxicity should lead to discontinuation of chemotherapy until recovery to at least grade 1. All uncertain cases of toxicities will be discussed by the study group discretion.

Patients can withdraw from study participation at any time. Patients are taken off the study if unacceptable toxicity appears. Unacceptable toxicity is defined as serious side effect or irreversible grade 4 toxicity, independent whether they are expected or unexpected. After the individual ending of the study subjects will receive the best available medical and nutritional care. Patients will undergo imaging and lab analysis (including tumor marker) quarterly and will be tracked by quarterly phone follow-up until death.

### Statistical consideration and sample size estimation

Three analysis sets are defined: the Intent-to-Treat (ITT) population including all patients who are randomized with study medication assignment designated according to initial randomization, regardless of whether patients receive study medication or receive a different medication from that to which they were randomized. This will be the primary population for evaluating all efficacy endpoints as well as subject characteristics.

The safety population consisting of all patients who received at least one dose of study medication. The per protocol population, consisting of all patients of the ITT population who completed at least one chemotherapy cycle, have at least 2 post-baseline assessments regarding the primary endpoint without pre-specified, selected major protocol deviations thought to impact on efficacy analysis.

#### Primary analysis

Stratification of patients regarding performance status prior to randomization may improve the power of the trial. For this reason the main analysis will be stratified by ECOG (stratum 1: PS < 2, stratum 2: PS ≥ 2) using the ITT population. A stratified log rank test will be used to compare the two treatment arms regarding the primary variable. The EFS-rates will be derived from the Kaplan Meier estimate and the confidence intervals will be calculated using Greenwood's formula. The above method relies on the assumption that events are recorded at the time they occur and there is no lag in the time at which they are reported. If this assumption is not satisfied, an alternative, interval censored analysis will be used.

#### Secondary analyses

A sensitivity analysis will be performed on the primary variable to assess the impact of protocol deviations using the per protocol analysis set.

The hazard ratio and the related 95% confidence interval (CI) for the experimental arm relative to control arm will be estimated by proportional hazard regression with treatment, centre and performance status as covariates.

OS, PFS, and TTP will be analyzed in the same manner as in the primary efficacy endpoint. Estimates of the ORR and 95% CI will be calculated and will be compared between treatment groups using the Cochran-Mantel-Haenszel (CMH) test adjusting by the stratification variables used in the stratified log rank test. For subjects with incomplete follow-up, time to last follow-up date will be used as the censoring time in the analysis of time-to-event data.

Descriptive statistics will be provided for the tumour-cachexia parameters. QoL scales, subscales and single item scores will be summarized by the mean and median for each arm and plotted by time. QoL data will be analysed using ANCOVA techniques. A logistic regression analysis will be carried out to identify relevant prognostic factors.

Incidence rates and 95% CI of Adverse Events (AEs) will be summarized. Subject disposition will be tabulated. Baseline characteristics, concomitant medications, vital signs and PS will be tabulated. Study drug administration and reasons for the deviations from the planned therapy will be tabulated. Summary tables will be prepared to examine the distribution of laboratory measures over time.

#### Interim analysis

One interim analysis is to be conducted when approximately 50% of the events have occurred. The nominal one-sided significance levels for the interim analysis are 0.0026 and 0.0240, respectively.

The objectives of the interim analyses are early stopping of the trial in case of extreme differences in the event rates while maintaining the overall two-sided significance level for the final analysis at 0.05 and assessing safety, including any unexpected toxicity.

All statistical tests will be performed at a two-sided significance level of 0.05 apart from the nominal one-sided significance levels of the interim analysis and final analysis determined by using the O'Brien-Fleming stopping rule.

#### Sample size estimation

The sample size is calculated to detect differences in the 4-month EFS-rates and is based on a comparison of two groups using the unstratified log rank test. The 4-month EFS-rates are assumed to be 60% and 40% (HR = 0.557) in the experimental and control group, respectively based on data from literature [6,7]. Applying one interim analysis according to group sequential design with two stages using the O'Brien and Fleming type, a 1:1 randomization, and assuming an accrual time of 36 and a follow-up time of 4 months a total of 102 patients and of 93 events are required for a two-sided log rank test with an overall two-sided significance level of 0.05 and power of 0.80 With the expectation that approximately 15% of patients may be lost to follow up, it is estimated that 120 patients will need to be enrolled. Formula of Schoenfeld [14] used for the calculation of the number of events. The ADDPLAN, Version 4.0 calculator was used for the sample size estimation. Stratification of heterogeneous patients regarding performance status prior to randomization may improve the power of the trial. We expect an additional stratification effect and in case of a smaller than the assumed true treatment effect the lost of power will be decreased. The primary analysis will be therefore performed with the stratified log rank test.

The statistical analysis will be carried out by the responsible biostatistician at the National Center for Tumor diseases (NCT) Heidelberg. The analysis will be done as soon as the database has been declared to be complete and accurate and has been locked. The details of the analysis will be laid out in the statistical analysis plan, which will be finalized and approved prior to the database lock. It has to be authorized before by the biometrician, the sponsor, and the Coordinating Investigator.

## Discussion

As outlined in the background section patients with advanced pancreatic carcinoma have to deal with several problems: (1) their life-expectancy is short; (2) they suffer from cachexia and deterioration in performance status [4]; (3) cachexia is a strong independent predictor of mortality, poor therapeutic response, diminished functional capacity, and reduced QoL; (4) pancreatic cancer has the highest incidence for cancer cachexia (CC), as much as 80% at the time of diagnosis [4], (5) cachectic patients are difficult to guide through chemotherapy experiencing more severe side effects resulting in progressive cachexia.

There are data indicating that the outcomes of subjects with CC receiving chemotherapy can be improved with PN [6]. Supplementation with PN improves the QoL in subjects with advanced CC [7]. There are also data indicating that PN has a synergistic effect with chemotherapy and results in better clinical outcome [6,7].

Unfortunately, most to nearly all clinical trials dealing with nutritional intervention enrol either patients with different tumours, and/or undefined oncological treatment and/or treat them with not precisely defined nutrition recorded often retrospective by the subjects in diaries. It is our aim to perform with PANUSCO a clinical trial which allows investigating as exactly as possible the impact of PN in cancer patients. To make the groups comparable we restrict the trial to subjects with histological confirmed pancreatic carcinoma, treat them with defined chemotherapy and randomize them to defined PN and BSNC or solely BSNC. As patients often do not stick to restrictions and/or instructions for oral intake we decided to allow every kind of oral or enteral supplementation and use PN to be sure about compliance in the experimental group.

## Conclusion

PANUSCO offers to our knowledge for the first time the chance to investigate the importance of NI in cancer cachexia in a clear defined setting. This might have implications for other entities and for supportive therapy ion oncology.

## Abbreviations

5-FU: 5-Fluorouracil; AE: Adverse Event; BIA: Bioimpedance Analysis; BSNC: Best Supportive Nutritional Care; CRF: Case Report Form; CRO: Contract Research Organisation; CTCAE Version 3.0: Common Toxicity Criteria Adverse Event Version; 3.0; DSMB: Data and Safety Monitoring Board; ECOG: Eastern Cooperative Oncology Group; EFS: Event-Free Survival; FA: Folinic Acid; GCP: Good Clinical Practice; IMBI: Institute of Medical Biometry and Informatics; MedDra: Medical Dictionary for Regulatory Activities; NCT: National Center for Tumor Diseases; NI: Nutritional Intervention; ORR: Objective Response Rate; OS: Overall Survival; PS: Performance Status; PINI: Prognostic Inflammatory and Nutritional Index; PN: Parenteral Nutrition; QoL: Quality of Life; SAE: Serious Adverse Event; SAP: Statistical Analysis Plan; SOP: Standard Operating Procedures; TTP: Time To Progression

## Competing interests

The authors declare that they have no competing interests.

## Authors' contributions

AM, MWB, DJ, CDB, IR, SH, MNW, and JS participated in the design of the study, IKS was responsible for the statistical planning of the trial, MNW and JO and wrote the study protocol. All authors read and approved the final manuscript.

## Pre-publication history

The pre-publication history for this paper can be accessed here:

http://www.biomedcentral.com/1471-2407/9/412/prepub
